# Clinical characteristics and cognitive function in bipolar disorder patients with different onset symptom

**DOI:** 10.3389/fpsyt.2023.1253088

**Published:** 2023-09-28

**Authors:** Zhonggang Wang, Haiyan Cao, Yuying Cao, Haining Song, Xianfei Jiang, Chen Wei, Zhenzhen Yang, Jie Li

**Affiliations:** ^1^Department of Psychiatry, Shandong Daizhuang Hospital, Jining, China; ^2^Institute of Mental Health, Tianjin Anding Hospital, Tianjin, China; ^3^Department of Medical Imaging, Affiliated Hospital of Jining Medical University, Jining, China; ^4^Department of Psychiatry, Jining Medical University, Jining, China

**Keywords:** bipolar disorder, clinical characteristics, cognitive function, first onset symptoms, risk factors

## Abstract

**Background:**

In recent years, studies on the clinical features and cognitive impairment of patients with different first-episode types of bipolar disorder have received increasing attention. The patients with bipolar disorder may present with different symptoms at first onset. The aim of this study is to assess the cognitive functions of a patient’s index episode of bipolar disorder, depression or mania, on risk factors of effecting on cognitive functions.

**Method:**

One hundred sixty eight patients with bipolar disorder diagnosed for the first time were enrolled in the study. All patients were divided into two groups according to their index episode of bipolar disorder, either depression or mania. Seventy three patients of the cohort had an index episode mania and 95 patients had initial symptoms of depression. Demographic and clinical disease characteristic data of all enrolled patients were collected. Meanwhile, 75 healthy controls were included. Demographic data of controls were collected. The cognitive functions of all patients and controls were detected by continuous performance test (CPT), digital span test (DST) and Wisconsin card sorting test (WCST). The main cognitive functions data were compared among the mania group, depression group and control group. The relevant risk factors affecting cognitive function were analyzed.

**Results:**

(1) Most patients with bipolar disorder had an index episode depression (56.55% vs. 43.45%). Compared with the depression group, the mania group had later age of onset [(24.01 ± 4.254) vs. (22.25 ± 6.472), *t* = 2. 122, *p =* 0.035]. The education level of patient groups was lower than control group (*p* < 0.001). (2) The healthy control group’s DST, WCST and CPT scores were better than the patient groups (All *p <* 0.05). The mania group’s DST (forward, reverse, sum), WCST (total responses, completed classifications, correct responses, incorrect responses, percentage of correct responses, completed the number of responses required for classification, the percentage of conceptualization level, the number of persistent responses, non-persistent errors), CPT (2 digit score, 3 digit score, 4 digit score) was better than the depression group (*p <* 0.05). (3) In mania group, correlation analysis showed that all CPT parameter, inverse digit span, and the sum of DST was negatively correlated with the education level (All *p <* 0.05). The CPT-4 digit score was negatively correlated with onset age (*p <* 0.05). In the WCST, the number of correct responses, the percentage of correct responses and the percentage of conceptualization level were positively correlated with the BRMS score (All *p <* 0.05). The number of false responses and persistent responses were negatively correlated with the BRMS score (All *p <* 0.05). The number of persistent errors and percentage of persistent errors was positively correlated with education years (All *p <* 0.05). In depression group, there was a positive correlation between inverse digit span and the education level (*p <* 0.05).

**Conclusion:**

In our study, there were cognitive impairments in attention, memory, and executive function of patients with different onset syndromes of bipolar disorder. Compared with the mania group, the degree of cognitive impairments in bipolar patients with the depressive episode was more severe. The risk factors affecting cognitive impairments included the age of onset, education level, number of hospitalizations and severity of illness.

## 1. Introduction

Bipolar disorder (BD) is a chronic and recurrent psychiatric disease affecting more than 1% of the population worldwide (1, 2). Bipolar disorder is characterized by a high prevalence rate, high disability rate, and high comorbidity rate (3, 4), that results in significant disability, heavy economic burden on patient and their families. Bipolar disorder occurs mainly in early adulthood, and most patients’ first onset is between 20 and 30 years old (4). Bipolar disorder is characterized by pathological changes in mood as well as recurring episodes of mania, hypomania, depression and mixed symptoms. The most common symptoms are manic episodes and depressive episodes, which manifests as manic symptoms clusters and depressive symptoms clusters.

Patients with bipolar disorder (BD) differ in their relative predominance of types of episode. Predominant polarity has predictive value in predicting group differences in course of illness. Compared to patients with depressive polarity, patients with manic polarity may represent a more distinct subgroup regarding illness course, suicide attempts, and psychiatric comorbidity (5). Colom et al. (6) thought depression was the dominant polarity both for bipolar I and bipolar II patients. Prevention of mania and depression would be equally important in the case of bipolar I patients. Prevention of depression is crucial for the maintenance treatment of bipolar II patients, whilst predominant polarity is a valid prognostic parameter with therapeutic implications. Most patients with bipolar disorder were found to have depressive episodes as their first symptom, and those with depressive episodes as their first type had a higher risk of suicide. Popovic et al. (7) found that patients with manic episodes as the dominant symptom were later diagnosed with bipolar I disorder, characterized by male predominance, earlier age of onset and first hospitalization, more hospitalizations, higher rates of substance abuse, and higher rates of concomitant psychotic symptoms; while patients with depressive episodes as the first symptom were later diagnosed with bipolar II disorder, with more depressive episodes, higher rates of stressful events, and higher risks of suicide attempts.

Some researchers had studied the distribution of onset type in bipolar disorder patients. Rangappa et al. (8) studied 285 bipolar I patients with a course of more than 5 years and found that manic episode was the main attack type (79%). The type of the first episode determined the main episode type in the lifetime episode, and the type of the first episode affected the treatment and prognosis of the disease. Patients with the first type of manic episode seem to have a better prognosis. For the distribution of first-episode types in bipolar disorder patients, Rangappa et al. (8) found that manic episodes were the predominant episode type (79%) in 285 patients with type I bipolar disorder with a disease duration of more than 5 years, that the first-episode type determined the predominant episode type throughout the future course of the disease, and that the first-episode type influenced the treatment and prognosis of the disease, with patients whose first-episode type was a manic episode appearing to have a better prognosis. The study of Baldessarini et al. (9) on 1,081 cases of bipolar disorder showed that the first symptom types of bipolar disorder were in the order of depressive episode (59%), manic episode (13%), episode with psychotic symptoms (8.0%), anxiety (7.6%), mild manic episode (6.7%), and mixed state (5.5%). Overall, depressive episodes as the first symptom were a more consistent finding in most studies, and patients with bipolar disorder with depressive episodes as the first symptom seemed to have a worse prognosis. According to Barcelona proposal for predominant polarity (10), 20.6% of the BD patients belonged to depressive predominant polarity, 45.8% belonged to manic predominant polarity and 33.6% belonged to indeterminate polarity. Those patients with depressive predominant polarity were more often having bipolar disorder II, had later age of onset, more time in episodes stage, more residual depressive symptoms, less residual manic symptoms, and so on.

Cognitive deficits are considered to be one of the core features of bipolar disorder, and the study of cognitive function in bipolar disorder is one of the current research hotspots (11, 12). Cognitive deficits predict the overall functional outcome of patients with bipolar disorder, and persistent cognitive deficits suggest a poor prognosis for patients with bipolar disorder (13). Cognitive deficits in patients with bipolar disorder are manifested in multiple domains, including attention, executive functioning, memory, intelligence, and judgmental and analytical skills (13).

Cognitive impairment has been found to exist not only during the acute onset of bipolar disorder (14, 15) but also during the remission phase of the disease (16, 17). Bora et al. (18) showed that deficits in neurocognitive development are already present in the early stages of bipolar disorder onset and that patients with bipolar disorder in the early stages of onset have significant neuropsychological deficits in IQ, verbal and deficits in IQ, verbal and visual memory, verbal fluency, and reasoning. However, individuals in the acute and clinically stable phases of bipolar disorder have deficits in different domains of cognitive functioning (including executive function, attention, memory, verbal comprehension, etc.) (19), suggesting that some of the indicators of cognitive deficits in bipolar disorder are state markers of the patient that change as the condition changes and some are genetic markers that remain relatively stable across the stages of change. Since bipolar disorder is highly familial (17, 18), it is entirely possible that some cognitive deficits exist in first-degree relatives of patients with bipolar disorder who do not have the condition (20). Therefore, exploring the clinical characteristics of patients with bipolar disorder and clarify the features of cognitive impairments is conducive to a more appropriate assessment the patients’ condition. It is beneficial to optimize the treatment method of patients with bipolar disorder.

In conclusion, BD patients commonly have cognitive deficits, and cognitive deficits affect the overall prognosis (21). Although cognitive function in BD patients has been studied extensively (22, 23), there is a paucity of literature examining whether cognitive impairment exists between BD patients with different first episode types, so our study focused on exploring this point. Based on previous findings, we hypothesized that BD patients with different first episode types has cognitive impairment during the stable phase of the disease and that there are differences in the characteristics of cognitive impairment, so we further clarified this issue by comparing the characteristics of cognitive impairment in BD patients with depressive symptoms as the first symptom and those with manic symptoms as the first symptom, in order to provide a comprehensive understanding of cognitive function in bipolar disorder patients, and provide a reference for better clinical intervention strategies.

## 2. Participants and methods

### 2.1. Study subjects

#### 2.1.1. Patients group

There were 168 BD inpatients recruited from Shandong Daizhuang Hospital in China between July 1, 2020 and June 30, 2021. Inclusion criteria were as follows: (1) primary diagnosis meeting the BD diagnosis based on International Classification of Diseases-10 (ICD-10) criteria, confirmed by two experienced psychiatrists; (2) Bech-Rafaelsen Mania Rating Scale (BRMS) ≤5 points for a minimum of 2 weeks; (3) Hamilton Depression Scale (HAMD) (24-item version) ≤7 points for a minimum of 2 weeks; (4) age 18–55 years old; (5) no gender limits; (6) having at least junior school education, understand the research project required in this study; (7) Han ethnicity, Chinese. Exclusion criteria were as follows: (1) diagnoses consistent with other psychiatric disorders in ICD-10; (2) patients with severe somatic diseases, including severe cardiovascular system diseases, immune system diseases, neurological diseases, etc.; (3) electroconvulsive therapy within 2 weeks; (4) alcohol, drug and various types of drug abuse; (5) female patients who are pregnant or breastfeeding; (6) disagree to participate in this study.

Manic episodes and depressive episodes are common types of bipolar disorder. All enrolled patients were divided into two groups according to the type of first episodes. One hundred sixty eight patients with bipolar disorder were divided into the first mania group (*N* = 73 patients) and the first depression group (*N* = 95 patients). These patients were treated with sodium valproate combined with olanzapine; antidepressant treatment was available as needed during depression episodes.

#### 2.1.2. Control group

The healthy controls group were staff, interns or registrars of our hospital during the same period, with gender and age matched to the patient group. A total of 75 healthy controls were enrolled, 44 males and 31 females, aged 18–55 years, with a mean of (25.31 ± 4.239) years.

The study protocol was reviewed and approved by the Ethics Committee of Shandong Daizhuang Hospital.

### 2.2. Research method

#### 2.2.1. Information collection questionnaire

A self-designed questionnaire was used to collect information on age, sex, occupation, family history, marriage, education level, and onset age in patients group. Information on age, sex, marital status, and years of education was collected from the health control group.

#### 2.2.2. Assessments of mental condition

1. Bech-Rafaelsen Mania Rating Scale (BRMS) (24).

The BRMS mainly assesses the severity of the subject’s manic symptoms. A score of 0 represents no symptoms or a level similar to the subject’s normal level, 1 represents mild symptoms, 2 represents moderate symptoms, 3 represents significant symptoms, and 4 represents severe symptoms. The total score reflects the severity of the disease, the higher the total score, the more serious the disease, 0–4 is no obvious manic symptoms, 6–10 is mild manic symptoms, ≧22 is severe manic symptoms.

2. Hamilton Depression Scale (24-item version) (HAMD-24) (25).

HAMD is the most commonly used scale to assess depressive symptoms in patients with psychiatric disorders in clinical practice. There are three versions of this scale, including 17-item, 21-item, and 24-item versions. The 24-item version was used in this study, and the 24 items were scored using a five-point scale from 0 to 4. 0-none, 1-mild, 2-moderate, 3-severe, and 4-very severe. Total score <8: normal; total score from 8 to 20: possible depression; total score from 20 to 35: definitely depression; total score ≧35: severe depression.

3. The Positive and Negative Syndrome Scale (PANSS) (26, 27).

The PANSS is mainly used to assess the presence or absence of psychiatric symptoms and the severity of each symptom in patients with psychiatric disorders. This scale was used in this study to assess the presence or absence of psychotic symptoms of the enrolled patients. Item scoring criteria: each item of PANSS has a clear definition and a clear 7-point scale, and the 7-point scales are: 1-none; 2-very mild; 3-mild; 4-moderate; 5-moderate-severe; 6-severe; and 7-very severe.

### 2.3. Cognitive measurement instruments

1. Wisconsin card sorting test (WCST).

The WCST is a neuropsychological test that reflects subjects’ cognitive functions such as neuropsychological processes, generalization, working memory, cognitive transfer, information extraction, attention, categorization switching, categorization maintenance, stimulus recognition and processing, sensory input and motor output. The test consists of 4 stimulus cards and 128 response cards. The WCST is suitable for measuring the cognitive function of clinical patients.

2. Measurements of digit span test (DST) (28).

The DST mainly measures subjects’ short term memory ability and attention. The DST consists of two parts, namely, parsimonious and backward memorization. The digit span parsimony mainly reflects the memory ability and attention ability of the subjects; the digit span reversal mainly reflects the executive functions of the subjects, especially working memory and cognitive flexibility.

3. Measurements of continuous performance test-identical pairs (CPT-IP).

**Figure 1:**
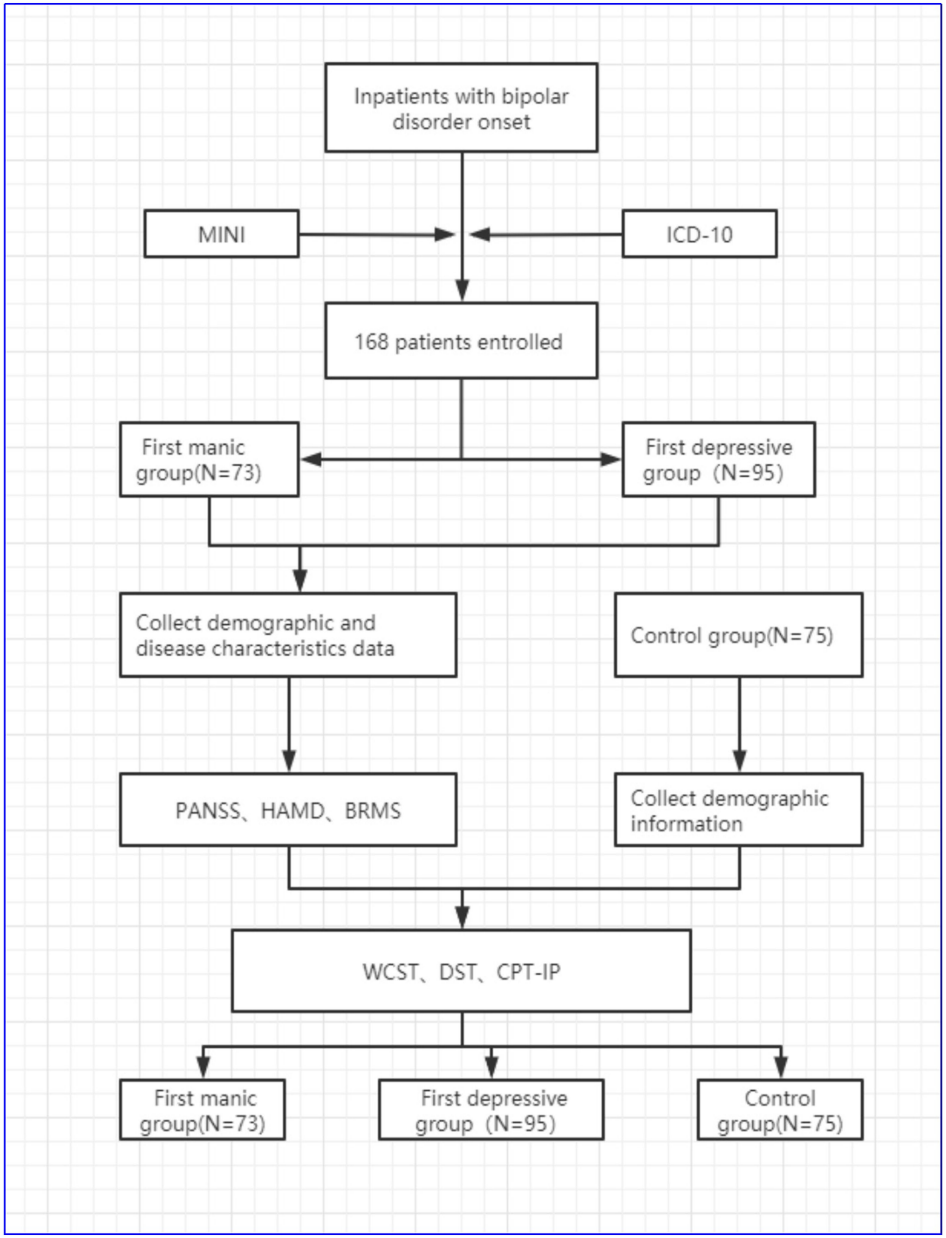
Research flowchart.

There are several versions of the CPT, and in this study, the CPT-IP was used to measure the sustained attention function in patients with bipolar disorder and healthy controls (see Figure 1).

### 2.4. Statistical analysis

All data were statistically analyzed using IBM SPSS version 20.0. For the measurement data such as age, age at first presentation, and other factors that conformed to normal distribution, they were expressed as (mean ± standard deviation), and one-way ANOVA was used for comparison between groups; those that did not conform to normal distribution were expressed as median (minimum, maximum), and non-parametric test (Kruskal–Wallis *H* test) was used. The chi-square test was used for gender, occupation, and other count data. The *χ*^2^ test was used to compare the differences in gender, occupation, family history, whether or not there were psychotic symptoms, whether or not there was a history of mental stimulation and marital status among the first manic group, first depressive group and normal control group, and the one-way ANOVA was used to analyze the differences in age and years of education among the first manic group, first depressive group and normal control group, and the independent sample *t*-test was used to compare the differences in first onset of mania and first depressive group. Non-parametric test was used to compare the differences in cognitive function scores among the first manic group, first depressive group and normal control group; Spearman’s correlation analysis was used to explore the correlation between cognitive function impairment and age at onset, years of education and number of hospitalizations in the first manic group and first depressive group. Test level *α* = 0.05, two-sided test.

## 3. Results

### 3.1. Demographic and clinical characteristics of bipolar disorder patients

A total of 168 patients were enrolled [73 (43.45%) in the first manic group and 95 (56.55%) in the first depressive group]. The details were showed in Table 1.

**Table 1:** Demographic and clinical characteristics of participants.

		[X̅ ± S or *N* (%)]
Age (years, mean ± SD)		27.26 ± 7.31
Onset age (years, mean ± SD)		23.02 ± 5.67
Admission times		3.48 ± 3.228
Education (years, mean ± SD)		11.43 ± 3.63
Episode mania group		*N* = 73 (43.45%)
Episode depression group		*N* = 95 (56.55%)
Sex (%)	Male	107 (63.7%)
	Female	61 (36.3%)
Occupation	Yes	105 (62.5%)
	No	63 (37.5%)
Family history of psychiatry disorder	Yes	54 (32.1%)
	No	114 (67.9%)
Psychiatric symptom	Yes	57 (33.9%)
	No	111 (66.1%)
Suspected precipitating event for first episode	Yes	46 (27.4%)
	No	122 (72.6%)
Marriage (%)	Married	60 (35.7%)
	Unmarried	90 (53.6%)
	Divorce	18 (10.7%)

### 3.2. Comparison of demographic and clinical characteristics among three groups

Compared with the first mania group, the first depression group had a higher percentage of patients with bipolar disorder (56.55% vs. 43.45%), the age of first onset [(24.01 ± 4.254) vs. (22.25 ± 6.472), *t* = 2.122, *p* = 0.035] was statistically significant between two patient groups, and the difference in education level among three groups was statistically significant (*p* < 0.001). The differences between the remaining indicators were not statistically significant (Table 2).

**Table 2:** Comparison of demographic and clinical characteristics among three groups.

	Mania group (*N* = 73, 43.45%)	Depression group (*N* = 95, 56.55%)	Control group (*N* = 75)	*F*/*χ*^2^/*t*	*p*-value
Sex				4.915	0.086
Male	53	41	44		
Female	20	54	31		
Age	26.67 ± 4.509	27.71 ± 8.877	25.31 ± 4.239	2.837	0.061
Onset age	24.01 ± 4.254	22.25 ± 6.472	—	2.122	0.035^*^
Admission times	2.95 ± 3.009	3.89 ± 3.344	—	−1.905	0.059
Education level	11.12 ± 3.537	11.84 ± 3.742	13.80 ± 4.094	10.970	<0.001^***^
HAMD score	2.74 ± 1.424	2.24 ± 0.953	—	2.575	0.011^*^
BRMS score	1.74 ± 0.898	1.62 ± 0.814	—	0.895	0.372
Occupation				5.507	0.064
Yes	50	55	56		
No	23	40	19		
Family history of psychiatry disorder			—	0.032	0.858
Yes	24	30			
No	49	65			
Psychiatric symptom			—	3.595	0.058
Yes	19	38			
No	54	57			
Suspected precipitating event for first episode			—	0.000	0.997
Yes	20	26			
No	53	69			
Marriage			—	7.130	0.129
Married	26	34	31		
Unmarried	44	46	36		
Divorce	3	15	8		

^*^p < 0.05, ^***^p < 0.001.

### 3.3. Comparison of sustained attention tests among three groups

The CPT scores of the first-episode mania group, first-episode depression group, and healthy control group did not conform to normal distribution, the median (minimum, maximum) was used to compare the CPT scores among the three groups using a nonparametric test (Kruskal–Wallis *H* test). The differences of CPT-2 digit score, CPT-3 digit score and CPT-4 digit score were statistically significant (all *p* < 0.001) among three groups (Table 3).

**Table 3:** Comparison of sustained attention tests among three groups.

	Mania group (*N* = 73, 43.45%) ①	Depression group (*N* = 95, 56.55%) ②	Control group (*N* = 75) ③	χ^2^	*p*	Mean rank
CPT-2	2.78 (2.12, 3.23)	2.23 (1.28, 2.80)	3.68 (3.00, 4.24)	76.249	<0.001^***^	③ > ① > ②
CPT-3	1.92 (1.30, 2.46)	1.49 (0.79, 1.87)	3.09 (2.36, 3.40)	82.941	<0.001^***^	③ > ① > ②
CPT-4	0.86 (0.59, 1.50)	0.50 (0.15, 1.11)	1.8 4 (1.28, 2.46)	77.218	<0.001^***^	③ > ① > ②

^***^p < 0.001.

### 3.4. Comparison of numerical breadth measures among three groups

A non-parametric test (Kruskal–Wallis *H* test) was used to analysis three groups of numerical breadth measures. The differences in DST-forwards, DST-backwards, DST-sum were statistically significant among three groups (all *p* < 0.001). The details were showed in Table 4.

**Table 4:** Comparison of numerical breadth measures among three groups.

	Mania group (*N* = 73, 43.45%) ①	Depression group (*N* = 95, 56.55%) ②	Control group (*N* = 75) ③	*χ* ^2^	*p*	Mean rank
DST-forwards	8 (7, 8)	7 (6, 8)	8 (8, 8)	31.125	<0.001^***^	③ > ① > ②
DST-backwards	4 (3, 4)	3 (2, 4)	5 (4, 5)	68.908	<0.001^***^	③ > ① > ②
DST-sum	11 (10, 12)	10 (9, 11)	13 (12, 13)	75.934	<0.001^***^	③ > ① > ②

^***^p < 0.001.

### 3.5. Comparison of WCST among three groups

There were statistically significant difference in total responses, number of completed categories, number of correct responses, number of incorrect responses, percentage of correct responses, number of responses required to complete the first category, percentage of conceptualization level, number of persistent responses, and non-persistent errors among three groups (all *p* < 0.05, or *p* < 0.001). Further comparison showed all above indicators of healthy control group were better than patients groups. These indicators of the first mania group were more better than the first depression group. Details were showed in Table 5.

**Table 5:** Comparison of WCST among three groups.

	Mania group (*N* = 73, 43.45%) ①	Depression group (*N* = 95, 56.55%) ②	Control group (*N* = 75) ③	*χ* ^2^	*p*	Mean rank
Total response	118 (96, 128)	128 (111, 128)	104 (85, 119)	35.030	<0.001^***^	② > ① > ③
Completed categories	6 (4, 6)	4 (2, 6)	6 (6, 6)	46.886	<0.001^***^	③ > ① > ②
Correct responses	67 (61, 75)	65 (52, 74)	72 (67, 80)	22.778	<0.001^***^	③ > ① > ②
Incorrect responses	37 (24, 64)	58 (37, 76)	26 (15, 36)	56.746	<0.001^***^	② > ① > ③
Percentage of correct responses	66.3 (50.39, 74.85)	55.47 (40.63, 66.67)	74.49 (68.57, 81.08)	57.247	<0.001^***^	③ > ① > ②
Number of responses required to complete the first category	16 (14, 21)	18 (12, 27)	15 (12, 20)	7.578	=0.023^*^	② > ① > ③
Percentage of conceptualization level	63.30 (42.97, 71.86)	45.31 (27.34, 62.86)	70.19 (63.64, 78.95)	54.182	<0.001^**^	③ > ① > ②
Persistent responses	20 (11, 38)	34 (19, 48)	11 (4, 17)	52.053	<0.001^***^	② > ① > ③
Persistent errors	4 (2, 8)	6 (2, 9)	4 (2, 9)	1.383	=0.501	—
Percentage of persistent errors	3.96 (2.35, 7.03)	4.69 (2.34, 7.14)	3.91 (2.20, 7.07)	0.087	=0.958	—
Non-persistent errors	29 (20, 59)	47 (27, 69)	21 (12, 26)	52.381	<0.001^***^	② > ① > ③

^*^p < 0.05, ^***^p < 0.001.

### 3.6. Correlation analysis of cognitive function scores and clinical characteristics in patients with bipolar disorder in the first onset manic group

Spearman’s correlation analysis showed the CPT-2 digit, CPT-3 digit, CPT-4 digit, DST-backwards, and DST-sum were negatively correlated with years of education, and CPT-4 digit score was negatively correlated with onset age (all *p* < 0.05). In WCST, the number of responses required to complete the first category was positively correlated with the number of admissions (all *p* < 0.05). The number of correct responses, the percentage of correct responses, and the percentage of conceptualization level were positively correlated with the BRMS score (all *p* < 0.05). The number of incorrect responses, the number of persistent responses, the number of persistent errors and the percentage of persistent errors were negatively correlated with the BRMS score (all *p* < 0.05). All details were showed in Table 6.

**Table 6:** Correlation between cognitive function scores and clinical characteristics of BD patients in the first onset manic group.

	Age (*r*/*p*)	Onset age (*r*/*p*)	Number of admissions (*r*/*p*)	Education level (*r*/*p*)	BRMS Score
CPT-2	−0.148/0.212	−0.094/0.429	−0.176/0.135	−0.241/0.040^*^	0.048/0.688
CPT-3	−0.233/0.047^*^	−0.142/0.232	−0.179/0.129	−0.239/0.042^*^	0.007/0.950
CPT-4	−0.227/0.054	−0.254/0.030^*^	0.011/0.924	−0.307/0.008^**^	−0.043/0.719
Correct responses	0.029/0.805	−0.134/0.259	0.076/0.524	−0.067/0.572	0.264/0.024^*^
Incorrect responses	0.083/0.483	0.120/0.312	0.013/0.915	0.173/0.142	−0.255/0.029^*^
Percentage of correct responses	−0.053/0.657	−0.116/0.328	0.029/0.809	−0.173/0.142	0.292/0.012^*^
Number of responses required to complete the first category	0.126/0.291	0.027/0.825	0.271/0.021^*^	0.084/0.482	0.085/0.480
Percentage of conceptualization level	−0.062/0.605	−0.140/0.239	0.042/0.726	−0.171/0.147	0.294/0.012^*^
Persistent responses	0.020/0.867	0.066/0.581	0.032/0.785	0.186/0.115	−0.294/0.012^*^
Persistent errors	−0.071/0.549	−0.180/0.128	0.072/0.543	0.320/0.006	−0.010/0.932
Percentage of persistent errors	−0.111/0.348	−0.184/0.120	0.015/0.896	0.295/0.011^*^	0.014/0.905
DST-backwards	−0.025/0.832	−0.001/0.992	−0.056/0.641	−0.296/0.011^*^	0.128/0.280
DST-sum	0.097/0.413	0.063/0.595	0.076/0.521	−0.235/0.045^*^	0.105/0.374

^*^p < 0.05, ^**^p < 0.01.

### 3.7. Correlation between cognitive function scores and clinical characteristics of bipolar disorder patients in the first onset depression group

Spearman correlation analysis showed DST-backwards was positively correlated with the level of education (*p* < 0.05). See Table 7 for details.

**Table 7:** Correlation between cognitive function scores and clinical characteristics of BD patients in the first onset depression group.

	Age (*r*/*p*)	Onset age (*r*/*p*)	Number of admissions (*r*/*p*)	Education level (*r*/*p*)	HAMD score
CPT-2	−0.063/0.542	−0.098/0.345	−0.184/0.075	0.095/0.362	−0.097/0.351
CPT-3	0.005/0.964	−0.017/0.871	−0.051/0.625	0.049/0.635	−0.094/0.365
CPT-4	0.032/0.755	0.006/0.956	−0.052/0.617	−0.010/0.922	−0.058/0.574
Correct responses	−0.043/0.682	−0.048/0.644	0.045/0.662	0.106/0.309	0.085/0.414
Incorrect responses	−0.081/0.437	−0.036/0.728	−0.119/0.250	−0.010/0.920	−0.039/0.707
Percentage of correct responses	0.129/0.212	0.005/0.964	0.094/0.366	−0.023/0.826	0.040/0.701
Number of responses required to complete the first category	−0.165/0.113	−0.092/0.380	0.003/0.979	0.109/0.297	0.047/0.656
Percentage of conceptualization level	0.139/0.180	0.029/0.782	0.144/0.164	0.004/0.972	0.041/0.693
Persistent responses	−0.134/0.194	−0.030/0.775	−0.065/0.534	0.023/0.827	−0.016/0.876
Persistent errors	0.062/0.551	0.000/0.999	0.085/0.413	−0.159/0.124	0.035/0.735
Percentage of persistent errors	0.070/0.501	0.010/0.926	0.104/0.316	−0.130/0.209	−0.001/0.993
DST-backwards	−0.063/0.547	−0.008/0.939	0.049/0.639	0.215/0.036^*^	−0.013/0.902
DST-sum	−0.086/0.410	0.029/0.781	−0.054/0.601	0.159/0.124	0.009/0.931

^*^p < 0.05.

## 4. Discussion

### 4.1. The basic clinical characteristics

Our study showed that the first symptoms of BD patients are mainly depressive episodes (56.55% vs.43.45%). Our previous study (29) supported this point. Compared with the first mania group, the first depression groups had more earlier onset age. There was a significant difference in education level among three groups. The educational level of both patient groups was lower than controls. Some researchers (30) explored the educational status of BD patients, and the study included 10,065 BD patients and found that only 38.96% of BD patients had a better educational level, and the educational level was worse in the presence of co-morbid substance abuse, anxiety disorders, and personality disorders. This research suggested that the disease possibly had a negative impact on patients’ educational level.

### 4.2. Attention function

Attention is the ability of selecting and integrating information, including think, remember, perceive, plan, execute, and so on. Sustained attention refers to the ability of an individual to focus on a task for an uninterrupted period of time, and sustained attention is a fundamental component of attention function, and the basis for higher-level attention and cognitive function. In our study, the attention function of BD patients with different first-onset types was measured by CPT. The data showed that the CPT-2 digit score, CPT-3 digit score, and CPT-4 digit score were higher in the healthy control group than the first-onset manic group, and higher in the first-onset manic group than the first-onset depressive group, suggesting that patients with different first-onset types of bipolar disorder have impaired attention function, and the degree of attention impairment in the first-episode depression group was greater than that in the first-episode mania group. All of these studies suggest that attentional abilities are significantly impaired in patients with bipolar disorder. Young et al. (31) explored the brain imaging mechanisms underlying impaired attention in bipolar disorder, and this study suggested that impaired attention in patients with bipolar disorder may be related to damage to the parietal cortex of the patient’s brain or inadequate expression of dopamine transporter proteins.

Studies on the variability of attentional deficits among patients with different first-onset types of bipolar disorder have not been reported. Previous studies (32–34) had examined the variability in the degree of attentional impairment between bipolar I disorder and bipolar II disorder. Kung et al. (32) found that sustained attention deficits were the common cognitive impairment in bipolar disorder patients, and almost all of information processing occurred during sustained attention. Fifty-one patients with bipolar disorder (22 with bipolar I disorder and 29 with bipolar II disorder) and 20 healthy controls were included in the study. The 17-item Hamilton Depression Inventory and Young’s Mania Inventory were used to assess the severity of the condition, and the continuous attention test was used to assess the subjects’ attentional function. It was found that after controlling the influences of severity, age, and education level, bipolar I disorder patients had longer delayed response times, poorer discrimination, and more misclassification errors than bipolar II disorder patients and healthy controls. It is suggested that bipolar I disorder patients have worse impairment in attention dysfunction.

### 4.3. Memory function

In our study, memory function impairment was found in both the first-onset mania group and the first-onset depression group. Memory function impairment was more severe in the first-episode depression group than the first-episode mania group, and the differences among three groups were significant for the three indicators of digit breadth compliance, digit breadth reversal, and digit breadth sum (all *p* < 0.001). There were not articles published on the memory function impairment in patients with different first-onset types of bipolar disorder. Otherwise, there were studies (35) perform executive functions and episodic memory in bipolar disorder. Cotrena et al. (35) found bipolar disorder type I was associated with more severe and widespread impairments than bipolar II disorder, which showed smaller impairments on all functions except inhibition, where impairments were larger. This study showed that patients with bipolar I disorder had more severe and extensive cognitive impairment compared to patients with bipolar II disorder; patients with bipolar II disorder had less impairment in cognitive functions except for inhibitory functions.

McKinney’s research (36) showed that patients with bipolar II depressive episode had impaired memory function in the early stages of the disease, and patients with psychotic symptoms had more worse memory function than healthy controls. Yatham et al. (37) showed that bipolar disorder patients had significant memory and attention impairment during depressive episodes, mostly in the form of memory loss and concentration difficulties. All studies suggested that bipolar disorder patients had cognitive functions impaired, including memory function, and there was different among episode types.

### 4.4. Executive function

Executive functions included generally planning, organizing, managing, working memory, response inhibition, emotional self-regulation, task initiation, and so on. On account of the executive function impairment in BD patients (38), so patients had a reduced ability to analyze and judge certain problems. The WCST was used to measure the executive function impairment in BD patients with different first-episode types. Studies of Eric et al. (39) and Kozicky et al. (40) showed cognitive impairment remains for bipolar disorder in stable or remission.

Schulze et al. (41) found BD patients and their first-degree relatives had abnormal executive function, mainly in response delay time. Therefore, Schulze (41) thought executive dysfunction in BD patients was related to genetic factors, and abnormal executive function may belong to the genetic endophenotypic markers of BD patients.

Impaired executive function in BD patients may be associated with multiple factors such as genetics and neural circuitry. Kulkarni et al. (42) thought abnormalities of frontotemporal lobes and subcortical loops were associated with executive dysfunction in BD patients. Newton thought (43) that abnormal levels of serum lipid peroxidation and brain-derived neurotrophic factor expressed were associated with impaired executive function in BD patients. Ni et al. (44) found MsrA haplotype may be associated with abnormal executive function. All studies showed that impaired executive function in BD patients was associated with certain genetic inheritance.

### 4.5. Factors affecting cognitive function

Our study showed three CPT digits, digit breadth backward and digit breadth sum were negatively correlated with education level (all *p* < 0.05), and the CPT-4 digit score was negatively correlated with onset age (all *p* < 0.05) in the first episode mania group. In WCST, the number of correct responses, the percentage of conceptualization level were positively correlated with BRMS scores (all *p* < 0.05). These findings suggested that the factors affecting the attention and memory functions of mania group patients included education level and onset age. Factors affecting the executive functions of mania group patients were mainly length of stay and education level.

This study showed there were relatively more disease characteristics correlated with cognitive impairment in BD patients with first episode mania. Szmulewicz et al. (45) found that neurodevelopmental abnormalities affecting on cognitive functional deficits. The metabolic syndrome has a negative impact on executive dysfunction of BD patients (46). The cognitive impairment in elderly BD patients was mainly related to the lower level of education and the higher incidence of medical diseases. Cognitive dysfunction increases disability and aggression in elderly BD patients (47). The genetic factors played an important role in the development of cognitive impairment in childhood BD patients (48). These studies suggest that there were many affecting cognitive impairment factors, and that the differences in cognitive impairment between patients with different types of first episode, and between different stages of the disease need to be studied further.

## 5. Conclusion

BD patients had multidimensional cognitive impairment, including attention, memory and executive function. Cognitive impairment of the first-episode depression group were more worse than the first-episode mania group. The factors affecting the cognitive function of BD patients education level, onset age, severity of illness, the number of admissions.

### 5.1. Limitations

The study was conducted entirely in one area of China, which may affect generalizability of findings. Another shortcoming of our study only considered the effects of drug types on cognitive function. We did not consider the effects of drug dosage on cognitive function. The method to determine the first episode rely on the patients history coming from the medical record or the patients or their relatives’ statement. We consulted patients or their relatives to identify the first episode type if it was not clear. We did not analyze the rapid cyclothymia type as a separate type, which is a shortness of this study. In addition, an assessment of what might be a protective factor of psychotherapy or counseling, often used prescribed in the prevention or mitigation of symptoms at onset, this point is not involved in our study. In future study, we will further improve the research design to make up for the above deficiencies.

## Data availability statement

The raw data supporting the conclusions of this article will be made available by the authors, without undue reservation.

## Ethics statement

The studies involving humans were approved by The Ethics Committee of Shandong Daizhuang Hospital. The studies were conducted in accordance with the local legislation and institutional requirements. The participants provided their written informed consent to participate in this study. Written informed consent was obtained from the individual(s) for the publication of any potentially identifiable images or data included in this article.

## Author contributions

ZW: conceptualization, formal analysis, writing—original draft, and supervision. HC: formal analysis, writing—original draft, and supervision. YC: formal analysis and writing original draft. HS: data curation and writing original draft. XJ, CW, and ZY: data curation. JL: conceptualization. All authors contributed to the article and approved the submitted version.
